# The feedback dilemma in medical education: insights from medical residents’ perspectives

**DOI:** 10.1186/s12909-024-05398-y

**Published:** 2024-04-19

**Authors:** Sara Shafian, Mehran Ilaghi, Yasamin Shahsavani, Maryam Okhovati, Adel Soltanizadeh, Sarah Aflatoonian, Ali Karamoozian

**Affiliations:** 1 Department of Medical Education, Education Development Center, Kerman University of Medical Sciences, Kerman, Iran; 2 Institute of Neuropharmacology, Kerman Neuroscience Research Center, Kerman University of Medical Sciences, Kerman, Iran; 3 Oral and Dental Diseases Research Center, Kerman University of Medical Sciences, Kerman, Iran; 4 Medical Informatics Research Center, Institute for Future Studies in Health, Kerman University of Medical Sciences, Kerman, Iran; 5 Department of Biostatistics and Epidemiology, Kerman University of Medical Sciences, Kerman, Iran

**Keywords:** Feedback, Medical residents, Medical education, Clinical training

## Abstract

**Background:**

Feedback is a critical component of the learning process in a clinical setting. This study aims to explore medical residents’ perspectives on feedback delivery and identify potential barriers to feedback-seeking in clinical training.

**Methods:**

This cross-sectional study involved 180 medical residents across seventeen specialties. We employed the validated Residency Education Feedback Level Evaluation in Clinical Training (REFLECT) tool to assess residents’ perspectives on their attitude toward feedback, quality of feedback, perceived importance, and reaction to feedback. Additionally, we explored barriers to feedback-seeking behavior among medical residents.

**Results:**

The majority of medical residents held positive attitudes toward feedback. They agreed that feedback improves their clinical performance (77.7%), professional behavior (67.2%), and academic motivation (56.7%), while also influencing them to become a better specialist in their future career (72.8%). However, the study revealed critical deficiencies in the feedback process. Only 25.6% of residents reported receiving regular feedback and less than half reported that feedback was consistently delivered at suitable times and locations, was sufficiently clear or included actionable plans for improvement. A minority (32.2%) agreed that faculty had sufficient skills to deliver feedback effectively. Moreover, peer-to-peer feedback appeared to be a primary source of feedback among residents. Negative feedback, though necessary, often triggered feelings of stress, embarrassment, or humiliation. Notably, there were no significant differences in feedback perceptions among different specialties. The absence of a feedback-seeking culture emerged as a central barrier to feedback-seeking behavior in the clinical setting.

**Conclusions:**

Establishing shared expectations and promoting a culture of feedback-seeking could bridge the gap between residents’ perceptions and faculty feedback delivery. Furthermore, recognizing the role of senior and peer residents as valuable feedback sources can contribute to more effective feedback processes in clinical training, ultimately benefiting resident development and patient care.

## Introduction

Teaching and learning in the clinical setting are integral components of medical education [1, 2]. Feedback delivery is critical to this learning process, enabling learners to understand what is expected from them and how to strive for excellence [3]. However, providing feedback in the clinical setting can be daunting due to the wide range of practical competencies and communication skills that need to be addressed, on top of considering the learner’s psychosocial needs while ensuring accuracy and honesty. Over the years, there have been changes in how feedback is perceived, as it is now seen as an ongoing dialogue between professors and students [4, 5]. This process describes the disparity between what is being performed by the student and what is expected of them [6].

Medical residency is a program through which individuals progress from general physicians to specialists [7]. Therefore, it represents a critical phase in which core competencies are developed. During these programs, the majority of learning takes place through work-based learning, case-based learning, problem-solving, and hands-on practical experience. As such, feedback is crucial to inform residents about their accomplishments and what needs improvement [8]. Improving the feedback delivery will ultimately impact the quality of patient care since the end goal is to train physicians who directly influence patient outcomes [9].

Before educators can address gaps and improve the quality of feedback they provide, it’s vital to assess the current status of feedback delivery. This is particularly crucial in clinical settings where learners juggle multiple theoretical and practical skills. Measuring the state of feedback delivery is an essential step towards advancing the learning process [10]. Although there are many styles and methods of providing feedback [11], there are also many barriers to effective feedback delivery [12, 13]. Because of the demanding and extensive hours medical residents are required to work, many of them express concerns that they lack adequate time to solicit feedback or that their instructors do not dedicate enough time to offer feedback to them. Moreover, many residents are apprehensive about giving and receiving feedback and feel anxious when meeting their supervisors to discuss their progress [14]. Some studies have found that residents prefer feedback that affirms their good performance rather than receiving feedback that criticizes their performance [15]. Furthermore, there have been reports that emphasize a disparity between the perceptions of professors and residents regarding feedback [16], revealing a lack of shared understanding of both the quantity and quality of feedback given.

In the context of medical education in Iran, the medical residency pathway follows a structured process. After completing an 18-month internship and graduating from medical school, physicians can apply for residency positions through a nationwide entrance examination. Successful candidates begin their specialized residency training, with program durations ranging from 3 to 5 years based on the specialty. During this period, residents undergo rigorous clinical training, gradually assuming increasing responsibilities under the supervision of attending physicians. Upon completing their residency, physicians are recognized as independent practitioners.

Considering the importance of feedback in clinical education, measuring and providing feedback can be a fundamental step for designing interventions to improve the delivery and receiving process. The overarching aim of this study was to comprehensively evaluate medical residents’ perspectives on the feedback delivery process during their clinical training. By understanding their attitudes, perceptions of feedback quality, perceived importance, and emotional responses, we sought to identify potential gaps and barriers in the feedback process. Ultimately, this understanding can inform strategies to optimize feedback delivery, thereby enhancing the learning experience and professional development of residents, which in turn can positively impact patient care.

## Methods

### Study design and participants

This cross-sectional study was performed at Kerman University of Medical Sciences (KMU), the largest medical school in southeast Iran. We included medical residents with at least six months from the beginning of their residency program at KMU. Taking into account the total number of registered residents at the time of study in KMU, the minimum required sample size was determined to be 175 based on the Morgan Table [17]. A total of 180 residents finally participated in the study. The number of participants from each specialty was determined using a quota sampling from each of the 17 specialty residency programs in KMU to ensure that a minimum of individuals in each specialty participated in the study. Participants were enrolled through direct contact during the clinical shift breaks, lecture breaks, departmental meetings or the conclusion of morning reports. Data was gathered through paper surveys by two of the research team members (medical interns at the time of study) who were not involved in the clinical training or supervision of the residents. This measure was taken to minimize any potential bias or coercion that could arise from a perceived power differential between the researchers and the residents.

To better compare different residency programs in terms of the feedback they receive, the specialties were categorized into three groups based on the general inherent characteristics of each residency program in Iran, including the surgical and hands-on competencies, number of on-calls and shifts, and weekly shift hours. According to these criteria, the first group consisted of minor specialties (group A), including the residents of radiation oncology, radiology, dermatology, neurology, ophthalmology, cardiovascular disease, psychiatry, pathology, and anesthesiology. It should be noted that using the term *“minor specialties”* does not imply any hierarchy or lesser importance but rather denotes a specific focus within certain clinical specialties in the Iranian context that are typically associated with lower weekly shift hours and fewer surgical procedures. The second group was major non-surgical specialties (group B), including the residents of internal medicine and pediatrics, and the third group constituted surgical specialties (group C) consisting of residents of general surgery, orthopedics surgery, urology, obstetrics and gynecology, otorhinolaryngology, and neurosurgery.

### Tools and measures

In order to assess the aspects of feedback delivery, we used the previously validated Residency Education Feedback Level Evaluation in Clinical Training (REFLECT) tool [18]. REFLECT is a 15-item questionnaire with a four-factor structure, including “attitude towards feedback” (items 1–5), “quality of feedback” (items 6–11), “perceived importance of feedback” (items 12 and 13), and “reaction to feedback” (items 14 and 15) that evaluates what medical residents’ perspective towards various aspects of feedback in clinical training is. Responses could be qualitatively reported based on participants’ choices or quantitatively scored according to a 5-point Likert scale (completely disagree = 0, completely agree = 4). The original version of the scale has been assessed in terms of content validity, test-retest reliability (Intraclass correlation coefficient = 0.949), and internal consistency (Cronbach’s alpha = 0.85). Moreover, a four-factor structure has been demonstrated according to the exploratory factor analysis [18]. Considering the importance of feedback-seeking behavior in clinical training, we additionally asked the participants through an open question if there were any barriers to feedback-seeking according to their perspectives. Accordingly, the participants were asked to indicate if there were any particular reasons preventing them from personally seeking feedback. Participants were allowed to indicate more than one reason. Responses were thematically analyzed by two independent researchers. Upon reaching a consensus, similar responses were categorized under the same themes, and the frequency of each barrier was reported.

### Statistical analysis

Data was analyzed using the Statistical Package for the Social Sciences (SPSS) software (version 26.0. SPSS, Inc., Chicago, IL, USA). For quantitative analysis, the mean and standard deviation (SD) were used to describe quantitative variables, and frequency and percentage were used to describe categorical variables. An analysis of variance (ANOVA) followed by a Tukey’s post-hoc test was used to compare the REFLECT questionnaire score among various field of specialty and according to the primary source of feedback. A p-value less than 0.05 was considered statistically significant. For qualitative analysis, the open-ended questions regarding barriers to feedback-seeking behavior, a thematic analysis approach was employed. Thematic analysis is a widely used qualitative method that allows researchers to analyze and report patterns or themes within the data. In this study, the responses to the open-ended question were gathered, and similar responses were categorized under the same themes by two independent reviewers. Subsequently, the reviewers compared and discussed their analyses to resolve any discrepancies and reach a consensus on the final themes. The frequency of each identified barrier was then reported, providing insights into the most prevalent challenges faced by residents in seeking feedback.

### Ethical considerations

This study has been conducted under the approval of the Ethics Committee of Kerman University of Medical Sciences (Ethics code: IR.KMU.REC.1400.646). To ensure voluntary participation, residents were informed that their involvement was entirely optional and that their decision to participate or not would have no bearing on their academic standing or future evaluations. Additionally, all participants completed the survey anonymously and no names were recorded.

## Results

A total of 180 medical residents participated in the study. The detailed demographic characteristics of participants are presented in Table 1. Females constituted 65.6% of the participants. The mean (± SD) age of residents was 31.0 (± 3.3). Most participants were PGY5 (28.9%), PGY3 (21.1%), and PGY2 (20.6%) residents. Among the residency programs in the studied population, internal medicine had the highest prevalence (18.3%), followed by obstetrics and gynecology (11.1%) and radiology (10.6%). The field of specialty was categorized as minor specialties (Group A), constituting 45.5% of participants; major non-surgical specialties (Group B) with 25.6% of participants; and surgical specialties (Group C), including 28.9% of studied medical residents (Table 1).

**Table 1:** Demographic characteristics of the study participants

Socio-demographic characteristics			N	Percentage %
Gender		Female	118	65.6
		Male	62	34.4
Postgraduate Year (PGY)		PGY 1	26	14.4
		PGY 2	37	20.6
		PGY 3	38	21.1
		PGY 4	27	15.0
		PGY 5	52	28.9
Specialty	Group A (*n* = 82)	Radiation Oncology	7	3.9
		Radiology	19	10.6
		Dermatology	8	4.4
		Neurology	11	6.1
		Ophthalmology	3	1.7
		Cardiovascular	13	7.2
		Psychiatry	13	7.2
		Pathology	5	2.8
		Anesthesiology	3	1.7
	Group B (*n* = 46)	Internal Medicine	33	18.3
		Pediatrics	13	7.2
	Group C (*n* = 52)	General Surgery	12	6.7
		Orthopedics Surgery	6	3.3
		Urology	5	2.8
		Obstetrics & Gynecology	20	11.1
		Otorhinolaryngology	3	1.7
		Neurosurgery	6	3.3
			Mean	Standard Deviation
Age			31.0	3.3

When asked about the frequency of feedback received from professors or colleagues, only 25.6% of the participants indicated that they receive feedback on a regular basis, while 73.3% indicated that they sometimes receive feedback. Senior residents were the primary source of feedback in 45.6% of the participants, followed by faculty (33.9%), and peer colleagues (20.5%) (Table 2).

**Table 2:** Residents’ perspectives regarding the frequency and source of received feedback

Item		N	Percentage %
Frequency of received feedback	Rarely	2	1.1
	Sometimes	132	73.3
	Regularly	46	25.6
Primary source of feedback	Faculty	61	33.9
	Senior residents	82	45.6
	Peer colleagues	37	20.5

Table 3 exhibits the distribution of participants’ responses to the REFLECT items. According to the responses in favor of an item (cumulative percentages of agree and completely agree responses), medical residents generally agreed that feedback improves their clinical performance (77.7%), professional behavior (67.2%), and academic motivation (56.7%), while also influencing them to become a better specialist in their future career (72.8%). Most residents (55%) considered their fellow or senior residents as a reliable source of feedback delivery. In terms of the feedback quality, only 38.3% indicated that feedback is provided at the appropriate time, and only 36.1% indicated that feedback is provided at the appropriate place. Less than half of the participants (48.9%) agreed that the provided feedback is completely clear. Intriguingly, only 41.1% of residents stated that they receive a solution along with the feedback. Overall, 31.1% admitted that the faculty spend sufficient time to provide feedback. Furthermore, only 32.2% of residents confirmed that the faculty have sufficient skills in providing feedback. Almost half of the residents (50.6%) admitted that they personally seek feedback if they do not receive sufficient feedback. Negative feedback was accompanied by a feeling of stress, embarrassment, or humiliation in 58.3% of residents. On the other hand, 94.4% of residents admitted that positive feedback results in a positive feeling (Table 3).

**Table 3:** Distribution of participants’ responses to REFLECT items

Item		Response N (%)				
		Completely Disagree	Disagree	No idea	Agree	Completely Agree
1	Feedback improves my clinical performance.	4 (2.2)	8 (4.4)	28 (15.6)	105 (58.3)	35 (19.4)
2	Feedback improves my professional behavior.	6 (3.3)	20 (11.1)	33 (18.3)	96 (53.3)	25 (13.9)
3	Feedback increases my academic motivation.	12 (6.7)	29 (16.1)	37 (20.6)	74 (41.1)	28 (15.6)
4	Feedback is influential in making me a better specialist in the future.	6 (3.3)	15 (8.3)	28 (15.6)	91 (50.6)	40 (22.2)
5	I consider my fellow or senior residents to be a reliable source for delivering feedback to me	13 (7.2)	32 (17.8)	36 (20.0)	81 (45.0)	18 (10.0)
6	Feedback is provided to me at the appropriate time.	10 (5.6)	48 (26.7)	53 (29.4)	53 (29.4)	16 (8.9)
7	Feedback is provided to me at the appropriate place.	15 (8.3)	47 (26.1)	53 (29.4)	53 (29.4)	12 (6.7)
8	The provided feedback is completely clear.	7 (3.9)	39 (21.7)	46 (25.6)	74 (41.1)	14 (7.8)
9	When receiving feedback, a solution is provided to improve my performance.	14 (7.8)	49 (27.2)	43 (23.9)	59 (32.8)	15 (8.3)
10	The faculty spend sufficient time getting to know me, evaluating me, and providing feedback.	33 (18.3)	53 (29.4)	38 (21.1)	41 (22.8)	15 (8.3)
11	In my opinion, the faculty have sufficient skills and follow an appropriate framework in providing feedback.	19 (10.6)	56 (31.1)	47 (26.1)	44 (24.4)	14 (7.8)
12	I consider the feedback from faculty to be necessary and important for my progress.	3 (1.7)	11 (6.1)	14 (7.8)	99 (55.0)	53 (29.4)
13	In case I do not find the received feedback sufficient, I personally seek feedback from professors or other residents.	8 (4.4)	42 (23.3)	39 (21.7)	75 (41.7)	16 (8.9)
14	Receiving negative feedback makes me feel stressed, embarrassed, or humiliated.	7 (3.9)	43 (23.9)	25 (13.9)	71 (39.4)	34 (18.9)
15	Receiving positive feedback makes me feel good.	3 (1.7)	0 (0.0)	7 (3.9)	103 (57.2)	67 (37.2)

Table 4 presents the calculated score for the total scale and the four subscales of the REFLECT questionnaire. No significant difference was observed in the total score and neither of the subscales among residents of different specialties, suggesting the relatively similar perspectives about feedback in residents of various fields. However, residents who most received the feedback from faculty, had a significantly higher score in the *“quality of feedback subscale”* compared to residents who most received feedback from peer colleagues (*p* = 0.044), implying that faculty generally provide higher quality feedback than peer resident fellows (Table 4).

**Table 4:** Quantitative analysis of scores obtained in REFLECT questionnaire and its subscales

Factor	Total Participants	Field of Specialty			Primary Source of Feedback		
		Group A	Group B	Group C	Faculty	Senior Residents	Peer Colleagues
Total Score	35.5 (± 9.2)	36.2 (± 9.3)	34.5 (± 7.6)	34.4 (± 10.5)	37.3 (± 9.6)	33.8 (± 9.5)	35.1 (± 7.5)
Attitude Towards Feedback	13.1 (± 4.0)	13.7 (± 3.8)	12.8 (± 3.7)	12.4 (± 4.5)	13.6 (± 3.8)	12.3 (± 4.5)	13.9 (± 3.2)
Quality of Feedback	11.8 (± 5.5)	12.2 (± 5.6)	11.2 (± 5.0)	11.9 (± 5.7)	13.3* (± 5.8)	11.4 (± 5.3)	10.5* (± 5.1)
Perceived Importance of Feedback	5.3 (± 1.6)	5.4 (± 1.7)	5.3 (± 1.2)	5.3 (± 1.7)	5.4 (± 1.5)	5.3 (± 1.7)	5.3 (± 1.4)
Reaction to Feedback	4.8 (± 1.2)	4.7 (± 1.24)	4.9 (± 1.1)	4.9 (± 1.3)	4.7 (± 1.2)	4.8 (± 1.2)	5.0 (± 1.13)

Data is expressed as mean (± SD)

*Significant difference (*p* < 0.05) between the groups according to Tukey’s post-hoc test

**Fig. 1:**
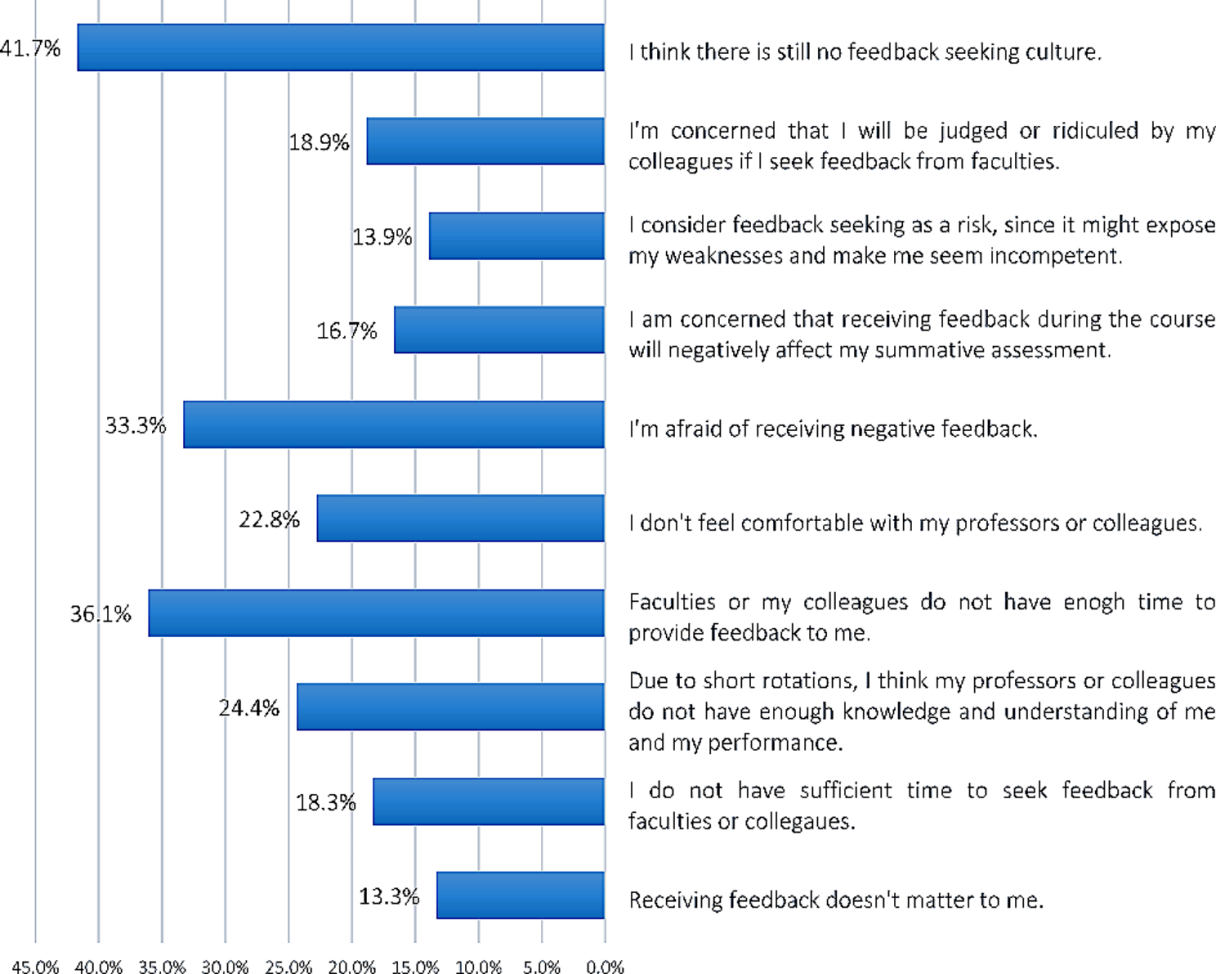
Barriers to feedback-seeking according to residents’ perspectives

We further asked medical residents what the major feedback-seeking barriers are. Figure 1 provides the barriers to feedback-seeking according to residents’ perspectives. Absence of feedback-seeking culture in the academic environment (41.7%), lack of time devotion from feedback providers (36.1%), and being afraid of receiving negative feedback (33.3%) were the most cited barriers (Fig. 1).

## Discussion

Feedback is crucial for medical residents as it helps them identify their strengths and weaknesses, improve their skills, and accomplish the required competencies. However, there is often a gap between their expected quality of feedback and what is provided. In this study, we strived to address this gap by asking the residents about various aspects of feedback and what the potential barriers to effective feedback delivery and feedback seeking might be.

The findings of this study showed that the majority of medical residents believed that feedback enhances their clinical performance, professional behavior, and academic motivation. Notably, our investigation revealed no significant disparities in residents’ attitudes towards feedback based on their respective specialties. This implies that the importance of feedback remains consistent across all specialties, irrespective of their unique characteristics. These findings underscore that residents view feedback as a constructive measure of their professional development. The same findings have been reported in other studies, indicating that residents have a positive attitude towards feedback in their clinical training [19]. This has also been reinforced by a recent systematic review and meta-analysis highlighting the feedback’s crucial role in improving learning outcomes and showing that those who received feedback tend to perform better regarding knowledge, attitude, and skills [20].

Although residents generally hold a positive view of feedback, we further highlighted several gaps in terms of both the frequency and quality of the feedback they receive. Our findings demonstrated that only 25.6% of the residents believed that they received regular feedback on their clinical performance, highlighting a substantial deficiency in the frequency of feedback. Moreover, according to the survey results, less than 40% of the respondents felt that feedback was given at the right time or in the right place. Furthermore, only 30% of them agreed that the faculty devoted sufficient time getting to know them and providing feedback. Findings from a separate study by Chao et al. also showed that inadequate time allocation and the absence of an appropriate setting of feedback delivery, mostly due to multiple clinical shifts and the busy schedules of faculty and residents, are some of the difficulties encountered when providing feedback [21]. Experts in medical education often advise that feedback should be given promptly after the learner’s observed performance to maximize its impact [22], suggesting that delaying feedback for too long after the performance can lessen its effectiveness and make it challenging for the instructor and resident to recall the details. Taken together, the findings of this research support the significance of professors allocating adequate time to offer timely feedback.

Our findings also showed that only a minority of residents (**≈** 32%) admitted that the faculty had sufficient skills to provide feedback. Moreover, several participants indicated that the feedback is not clear or does not contain a solution or practical action plan to improve their performance. Generally, a significant challenge of feedback delivery in the clinical setting is the lack of mutual understanding between the faculty and the learner regarding the feedback. For instance, a study by Sender Liberman and colleagues demonstrated a significant difference in the perception of feedback between the residents and professors. Accordingly, they reported that while professors felt that they almost always gave concrete suggestions for improvement and felt that they were successful at giving effective feedback, the majority of residents did not agree [16]. Therefore, while our study suggests that the quality of feedback might not be adequate enough according to residents’ perspective, this might not be what the faculty think about the feedback they provide. As a result, it seems crucial to hold shared sessions to clarify the expectations of both residents and faculty in providing and receiving feedback, thus promoting mutual and shared understanding.

Our findings also demonstrated that senior residents were the primary source of feedback for 45.6% of the participants, indicating that faculty should not be considered the sole providers of feedback in clinical training. Moreover, more than half of the residents believed that their fellow or senior residents were reliable sources of providing feedback to them. This finding is critical since residents usually spend most of their clinical experiences with their senior or peer fellows during their clinical shifts. Therefore, peer colleagues must be recognized as potential feedback providers. The significance of peer-to-peer feedback has been highlighted in several previous studies. A study by de La Cruz et al. discovered that a majority of medical residents found peer-to-peer feedback an essential component of their clinical training, suggesting that senior and fellow residents are valuable feedback sources in clinical training [23]. This finding has gone beyond a specific profession, as van Schaik and colleagues demonstrated that students have positive perceptions of inter-professional peer-to-peer feedback [24]. Furthermore, another study has suggested that peer observation of residents during work rounds resulted in an increased inclination of residents to give feedback to peers while also making them more comfortable receiving feedback from their peers [25]. Overall, considering that peer-to-peer feedback constitutes a significant portion of feedback the residents receive during their daily training, educating them on providing effective feedback and promoting a culture of peer observation, could make a considerable impact on the quality and quantity of feedback among residents.

The emotional response of the learner is a key factor to be considered when providing feedback [26]. Therefore, we further assessed the reaction to feedback among medical residents. Expectedly, almost all participants stated that receiving positive feedback results in a good feeling. On the other hand, more than half of the respondents declared that negative feedback makes them feel stressed, embarrassed, or humiliated. The negative emotional response towards negative feedback might adversely affect the feedback provided. According to a recent study by Koch et al., trainers may avoid giving negative feedback to their learners due to fear of adverse reactions [27]. Moreover, another study by Mitchell and colleagues suggests that avoiding providing negative feedback may stem from a desire to maintain a good working relationship with the learner or a fear of negative responses [28]. Recent research by Erickson et al. also indicates that negative emotional language used in feedback may cause recipients to focus on their emotions instead of responding positively [29], suggesting that the feedback provider should consider how expressions of emotion accompanying feedback affect attention and further performance of the learner. To reduce the emotional burden of negative feedback, several feedback frameworks, including sandwich feedback or the Pendleton method are suggested [30]. Furthermore, maintaining a positive teacher-learner relationship can also lead to a more positive reception of feedback.

We ultimately assessed the barriers to feedback-seeking behavior among medical residents. Although our findings indicated that feedback seems essential to the majority of residents, only half of them admitted that they personally seek feedback in case they do not receive sufficient feedback. We showed that the absence of feedback-seeking culture, lack of time devotion from feedback providers, and being afraid of receiving negative feedback are among the most important barriers to feedback-seeking in the clinical environment. Similarly, in a separate study, Delva et al. suggested that feedback-seeking in medical residents is dependent on four central factors: learning/workplace culture, relationships, purpose/quality of feedback, and emotional responses to feedback [13]. They proposed that enhancing the workplace and learning culture through longitudinal experiences, the implementation of structured feedback forms, setting explicit expectations for residents to actively seek feedback, while fostering a sense of safety and allowing sufficient time for observation and feedback provision, could effectively promote feedback-seeking behavior among residents. Residents generally believe feedback should be a regular part of their educational and work environments. However, because clinical work mostly takes priority over learning, they hesitate to ask for feedback. Therefore, promoting a culture of feedback-seeking within the clinical environment seems crucial.

This study had several strengths and limitations. Our study benefits from a reasonable number of medical residents representing various specialties, allowing for a comprehensive examination of feedback delivery perspectives. Furthermore, the utilization of a previously validated tool where key stakeholders (including medical education instructors and faculty) were involved in the development and validation process enhanced the credibility of the assessment. We additionally, identified barriers to feedback-seeking behavior, providing valuable insights for improving the feedback process in clinical training. However, the study’s reliance on self-reported data may introduce response bias, and the single-center design limits the generalizability of findings to other institutions. Finally, in this study, our examination was limited to the assessment of residents’ perspectives regarding feedback. However, it is imperative that future investigations broaden their scope to encompass the viewpoints of faculty members as well. This inclusion is pivotal, as faculty perspectives constitute a complementary and vital facet of the overall narrative and their insights are essential in formulating strategies for enhancing the delivery of feedback.

## Conclusions

In conclusion, this study underscores the paramount importance of feedback in medical residency training, with the majority of residents recognizing its positive impact on their clinical performance, professional development, and academic motivation. Despite these positive attitudes, several critical deficiencies in the frequency and quality of feedback were identified, emphasizing the need for improvements in feedback delivery. The significant role of senior and peer residents as feedback providers suggests the potential for fostering a culture of peer-to-peer feedback. Addressing the barriers to feedback-seeking behavior, such as the lack of a feedback-seeking culture and fear of negative feedback, is crucial to further enhance the feedback process in clinical training. These insights can guide interventions aimed at optimizing feedback delivery, ultimately benefiting the professional development of medical residents and, by extension, the quality of patient care.

## Data Availability

The datasets used or analyzed during the current study are available from the corresponding author upon reasonable request.

## Abbreviations

KMU: Kerman University of Medical Sciences
REFLECT: Residency Education Feedback Level Evaluation in Clinical Training
SD: Standard Deviation
PGY: Postgraduate Year
